# Investigating the level of satisfaction of policyholders with supplementary health insurance in Iran

**DOI:** 10.1186/s12913-023-10134-1

**Published:** 2023-11-11

**Authors:** Asma Hamzeh, Nasrin Hozarmoghadam, Mitra Ghanbarzadeh

**Affiliations:** Insurance Research Center, Tehran, Iran

**Keywords:** Supplementary health insurance, Policyholders' satisfaction, SERVQUAL model

## Abstract

The importance of customer satisfaction in business prosperity is undeniable, so many organizations consider customer satisfaction as the main driver of their business growth and try to keep their customers satisfied. The business market has never been so competitive in most areas. This is the reason why things like customer experience and customer loyalty are more and more important and are considered an indicator to measure the success of the business. Based on this, in this research, we will examine the level of satisfaction of insurance policyholders with supplementary health insurance services in Iran using the SERVQUAL model. This model is one of the most common models used in the field of quality assessment in the service sector. The method of conducting this research is a descriptive survey. The sampling method in the current research is simply random the statistical population is insurance policyholders and the sample number is 686 people from the society. The findings of the current research showed that there is a negative and significant gap in all dimensions of service quality.

## Introduction

Supplementary health insurance is a type of insurance from the subsets of personal insurance and is additional coverage to basic health insurance. This insurance covers various costs of medical and treatment services, such as hospitalization, outpatient surgery, medicines, and the cost of illness, which is partially covered by social security insurance.

The noteworthy point is that in today's competitive world and considering the multitude of different insurance companies, customer satisfaction is the most important factor of competition among companies. For this reason, knowing the level of customer satisfaction is very important for managers [5].

Insurance companies are not exempted from this rule, especially for insurance companies active in the field of treatment, due to the relevance of this field to medical, therapeutic, general health, and human life, the issue of customer satisfaction and measuring service quality is of particular importance.

Also, among the effects of customer satisfaction, we can mention improving the company's reputation and image, reducing changes in customers, increasing attention to customer needs, reducing costs, and finally increasing employee satisfaction and workforce stability. Knowing the company's customers and knowing their buying behavior creates a competitive advantage for companies. Retaining existing customers is more important than attracting new customers in the health insurance business. Because the cost of keeping an existing customer is lower than the cost of marketing to a new customer. Therefore, the satisfaction of policyholders in different fields of insurance should be examined, and the field of medical insurance, which deals with people's health and life, is very important.

A number of studies have investigated the level of satisfaction of insurance policyholders in different fields of insurance, which are briefly reviewed below.

Fatimah et al. [6], compared patient satisfaction when using the insured and non-insured in Public Health Center (Puskesmas Kasihan 1) Bantul, Indonesia. This study was a cross-sectional study, with 222 samples with 111 respondents using health assurance and 111 non-assurance. Samples were collected with a cluster sampling technique taken from nine service polyclinics. Data were analyzed using an independent sample t-test. Wray et al. [14] in their paper, studied the experiences of people with public and private health insurance among the 5 major forms of coverage in the United States. This survey study used data from 2016 to 2018 from the behavioral risk factor surveillance system on 149,290 individuals living in 17 states and the District of Columbia, representing the experiences of more than 61 million American adults. They found that the average patient satisfaction with health insurance was 34.76 more than the average patient satisfaction with non-health insurance was 29.10.

Geng et al. [7], in their paper, established a hypothetical model that comprised patients’ awareness of insurance policies, the fulfillment of patients’ expectations of insurance benefits, patients’ perceived value of health insurance coverage, patients’ satisfaction with health insurance programs, patients’ complaints, and trust in health insurance programs. They performed a confirmatory factor analysis by using a structural equation modeling (SEM) approach to examine the hypothesized model. A model-testing survey in 10 tertiary hospitals was conducted between June and October 2018, with a valid sample of 922 insured patients with chronic diseases. Their findings highlighted the importance of incorporating patients' perceived value as part of the ongoing efforts to increase satisfaction with health insurance by patients, especially those who have chronic diseases. Policymakers are also suggested to formulate evidence-informed reimbursement policies that meet patients' expectations.

Sarker et al. [11], conducted a cross-sectional survey of households around a pilot health insurance program during the months of April to June 2014 to compare and evaluate healthcare services in Bangladesh. Their study observed that the overall satisfaction level towards health services is quite favorable, but satisfaction scores can still be improved. These findings could contribute towards developing and designing the healthcare services packages of a community-based health scheme that is in line with the healthcare financing strategy of Bangladesh as well as the recommendation of the World Health Organization for developing social health insurance as part of the path to Universal Health Coverage.

Sogunro and Abiola [12] carried out to measure the buyers' trade-offs among multi-attributed products and services (utilities) that are derived from purchasing a particular life insurance plan using descriptive statistical analysis (Mean Score). The result showed that the policyholders generally are not satisfied with the Life insurance products based on the attributes attached to each of the products.

Dehghani et al. [5], in their research, investigated factors affecting the insured’s satisfaction with life insurance with the use of SERVQUAL Model in Novin Insurance Company. The research results demonstrated that in four dimensions of SERVQUAL model, there exists a gap between the expected quality and the insured’s understanding of the quality of services and that the largest gap is related to empathy, and the smallest gap is related to reliability.

Jadoo et al. [2], have been a cross-sectional study titled determining the level of patient satisfaction with the National Health Insurance in Istanbul (Turkey) in order to determine the level of patient satisfaction and identify the factors affecting satisfaction with the recently reformed National Health Insurance of Turkey. In bivariate chi-square analysis, the result of this study indicated that there were eight factors significantly associated with the level of satisfaction i.e. age, gender, marital status, education, occupation, self-perceived health status, area of residency, and type of household's plan. Further analysis, by using multiple logistic regression showed that the eight factors are also significant predictors of the level of satisfaction. Higher patients satisfaction was associated with improved access to care and continuity of care. However, light must be shed on the availability of resources, technical quality, and humaneness to improve overall patients satisfaction.

Gulati et al. [7], in research, investigated the level of customer satisfaction in the medical insurance industry of India, and in it, the quality of services perceived and expected by insurance customers in this country was determined from a questionnaire that was made based on the SERVQUAL model, they evaluated. The results of this study showed the major differences in customers’ expectations and perceptions of insurance services and as a result, showed dissatisfaction among insurance company customers.

Anvary Rostamy [1], investigated the factors affecting the quality of bank services and the gaps in the perceptions and expectations of customers and employees regarding the quality of bank services. To collect data, a customized SERVQUAL questionnaire was prepared and distributed among 385 customers and 305 employees. The results showed a significant difference between the views of customers and employees.

This research aims to measure the level of satisfaction of policyholders with supplementary health insurance and analyze the results so that managers can use the results obtained in their decisions and take the necessary measures to improve the quality of their services and maintain their customers.

## Theoretical foundations and backgrounds

During the last few decades, service quality has become one of the main areas of attention of managers and researchers. The reason for this attention is the effect of service quality on cost reduction, customer satisfaction, customer loyalty, profitability, and performance improvement. A lot of research has been done on the definition, modeling, measurement, data collection method, data analysis, etc., in the field of service quality and to measure satisfaction [1].

In today's markets, the customer is the guarantor of the organization's survival. What the customer wants has value, and the organization must take steps toward the customer's wishes. Customers also find value in high quality at a reasonable price. Customers are the lifeblood and one of the most valuable assets of the organization, and without them, the performance of the organization will be meaningless and there will be no organizational jobs [9].

The different models and patterns presented by quality management science researchers and economic and marketing scientists to measure customer satisfaction can be divided into two categories, which are described below:

- ➢ Objective methods: These methods indirectly measure customer satisfaction by measuring indicators that have a strong correlation with customer satisfaction. Due to doubts about the validity and accuracy of these methods, these methods are used less.
- ➢ Theoretical or conceptual methods: In these methods, customer opinions are directly used to measure customer satisfaction. Therefore, these methods are more valid than objective methods. The theoretical or conceptual methods are divided into two categories: characteristic and event-oriented methods [10].

Customer satisfaction is a hidden and qualitative variable, and to convert it into a measurable quantity, we need a suitable model and algorithm. With the help of a good algorithm and model, customer satisfaction can be converted into a numerical index and the factors affecting it can be measured quantitatively. In the last decade, several models have been proposed for customer satisfaction, including Kano model, SCSB model, ACSI model, and SERVQUAL model.

The SERVQUAL model is a mental model for measuring customer satisfaction with a company's services; In other words, this model works based on customers' perception of their satisfaction. The model directly uses the opinions of customers and presents an image of their satisfaction or dissatisfaction. The SERVQUAL model is one of the models that consider the perception and opinions of customers to measure the quality of services and is one of the most important models for measuring the quality of services It is widely used worldwide.

**Fig. 1:**
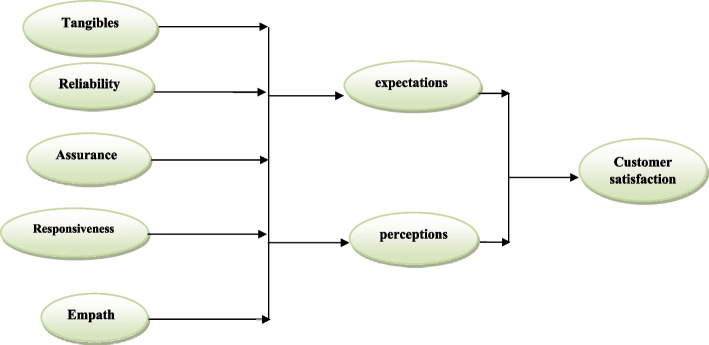
SERVQUAL model

The service quality measurement tool was presented in 1985 by Parasuraman and his colleagues to measure service quality and was revised and modified in 1991, 1988, and again in 1994. The five dimensions of SERVQUAL are actually a summary of the most important criteria that customers use when evaluating the quality of services. This tool is presented to measure the quality of services received in the form of 22 pairs of questions graded using a Likert scale. The first 22 options are designed to evaluate the expectations of customers from services and the next 22 options are designed to measure the perceived level of received services (perceptions). The SERVQUAL model is a mental model for measuring customer satisfaction with the company's services; In other words, this model works based on customers' perception of their satisfaction. This model directly uses the opinions of customers and presents an image of their satisfaction or dissatisfaction. The SERVQUAL model is one of the models that take into account the perceptions and opinions of customers to measure the quality of services, and it is one of the most important models for measuring the quality of services that are widely used worldwide. Based on this service-quality model, researchers have identified five determinants of service quality (see Fig. 1), which include: Tangibles, Reliability, Responsiveness, Assurance, and empathy [13].

The main reason for choosing the SERVQUAL model in this article is to pay attention to the desirable features of the SERVQUAL model, which will be presented below:

1. Periodic and consecutive use of SERVQUAL tool makes a company get closer to its desired result, which is "customer's understanding of service quality", and gain the ability to compare itself with other competitors.
2. The use of the SERVQUAL tool provides the opportunity for a company to evaluate its service quality performance based on each dimension both individually and as a whole.
3. Using the SERVQUAL model allows the company to classify its customers into different segments based on the obtained scores and to design different programs for each group to create more satisfaction in the future.
4. The approach to analyzing the SERVQUAL gap is a logical and straightforward concept, and the questionnaire has been explained in advance and can be easily adjusted if necessary.
5. Finally, it should be said that the SERVQUAL model is a test tool that can be used for marketing purposes in companies due to the use of a valid statistical tool and the possibility of updating and comparing the results after each SERVQUAL execution.

## Data and methodology

In this research, a questionnaire and a SERVQUAL model were used to obtain data.

The service quality dimensions of SERVQUAL have considered independent variables and customer satisfaction with service quality is the dependent variable.

The hypotheses of the current research, which were formed based on the conceptual model of SERVQUAL, include one main hypothesis and five sub-hypotheses. The sub-hypotheses correspond to the five dimensions of the SERVQUAL model. These hypotheses are as follows: The main hypothesis is: Customers are satisfied with the quality of supplementary health insurance services provided by insurance companies in Iran.

Sub-hypotheses:

- Customers are satisfied with the tangible dimensions of insurance companies in Iran.
- Customers are satisfied with the reliability of insurance companies in Iran.
- Customers are satisfied with the responsiveness of the employees of insurance companies in Iran.
- Customers are satisfied with the assurance of insurance companies in Iran.
- Customers are satisfied with the empathy of employees of insurance companies in Iran.

In this research, a completely random sampling was done among policyholders of supplementary health insurance in Iran in 2022. The data was collected through a questionnaire and analyzed using structural equation modeling and statistical methods.

The statistical population included all policyholders of supplementary health insurance in Iran. Therefore, the size of the population is very large. If the size of the population is very large, Cochran's formula for indeterminate communities is used as follows [4].

$$\begin{array}{c}n=\frac{{t}^{2}pq}{{d}^{2}}\\ d=0.05, t=1.96,p=0.5,q=.5\\ n=384\end{array}$$

Six hundred eighty six people responded to the questionnaire, which according to the above formula is sufficient.

The data collection tool in this study was a questionnaire consisting of two parts. The first part contains questions about people's demographic information, and the second part contains questions to measure the quality of services provided, which measures the perceptions and expectations of the customers. For this purpose, SERVQUAL standard questionnaire was used and with the help of factor analysis and SPSS software, a scale was designed for SERVQUAL service quality dimensions. Necessary care has been taken in designing the questions of the questionnaire so that the questions have enough simplicity and clarity.

Content validity was used to test the validity of the questions. To measure the validity of the content of the questionnaire, the opinions of specialists and expert experts were used. At this stage, by conducting various interviews and obtaining the opinions of the mentioned people, the necessary corrections were made and thus it was ensured that the questionnaire measures the same characteristic that the researchers wanted. Also, in order to determine the reliability of the questionnaire, Cronbach's alpha coefficient was calculated. It was equal to an acceptable value of 0.80.

SPSS version 26 statistical software was used in the statistical analysis in this research.

## Results

In order to analyze the descriptive statistics of the questionnaire, first, the demographic characteristics of the respondents, have been examined. The results are shown in Table 1. The statistical population included all policyholders of supplementary health insurance in Iran, and 686 people responded to the questionnaire.

**Table 1:** Demographic characteristics of the sample

Variable	Percents in the Groups	
Gender	Male: 22.30%	Female:77.70%
Marital Status	Married: 79:20%	Single: 20.80%
Level of Education	Diploma and below: 10.80%, Master: 31.80%, Associate: 8.70%, Bachelor: 39.90%, PhD and above: 8.70%	
Employment Type	Employed in public: 28% Self-employed: 8.60% Housewife: 1.50% Employed in private: 55.80% Student: 1.60% Retired: 0.60% Other: 0.90%	

About 19.80 percent of the respondents have one dependent (covered by insurance through insured) in health insurance, 25.4 percent of the respondents have 2 dependents, 20% of them have 3 dependents, 10.5% of them have 4 dependents, about 5.20% have 5 or more than 5 dependents and 19.1% of them have no dependents in health insurance.

Also, about the number of years that respondents have the health insurance, maximum percent is related to first year of coverage (52.60%) and minimum percent is related to 4 years of coverage (7.90%).

After conducting the descriptive statistics of the questionnaire results, we proceed to the inferential analysis of the obtained information.

Once participants expressed their perception of the services provided, and in this way, the perception scores were obtained. Once again, they were asked to express their expectations of the service that should be provided, and in this way, the scores of expectations were also obtained. The service quality gap was obtained from the difference between the scores of perceptions (the current state of service quality) and the scores of expectations (the desired state of service quality). If the score is positive, it means that the service provided exceeds the expectations of the customers, and if it is negative, it means that the service does not meet the expectations of the customers and there is a gap in quality, and if the score is equal to with zero, it means that there is no gap in quality, which indicates that the services provided to customers are within the expected range. Regarding the current questionnaire, the score was negative, which means there is a gap in service quality.

In this section, confirmatory factor analysis has been used to check to construct validity. The most important goal of confirmatory factor analysis is to determine the power of the predefined model with a set of observed data. Confirmatory factor analysis seeks to determine whether the number of factors and variable loadings measured on these factors is consistent with what was expected based on the theory and theoretical model. In other words, factor analysis tests the degree of conformity between the theoretical structure and the experimental structure. Here, factor analysis is used to investigate whether the five dimensions of the SERVQUAL model are loaded on the satisfaction variable, as predicted by the model.

**Fig. 2:**
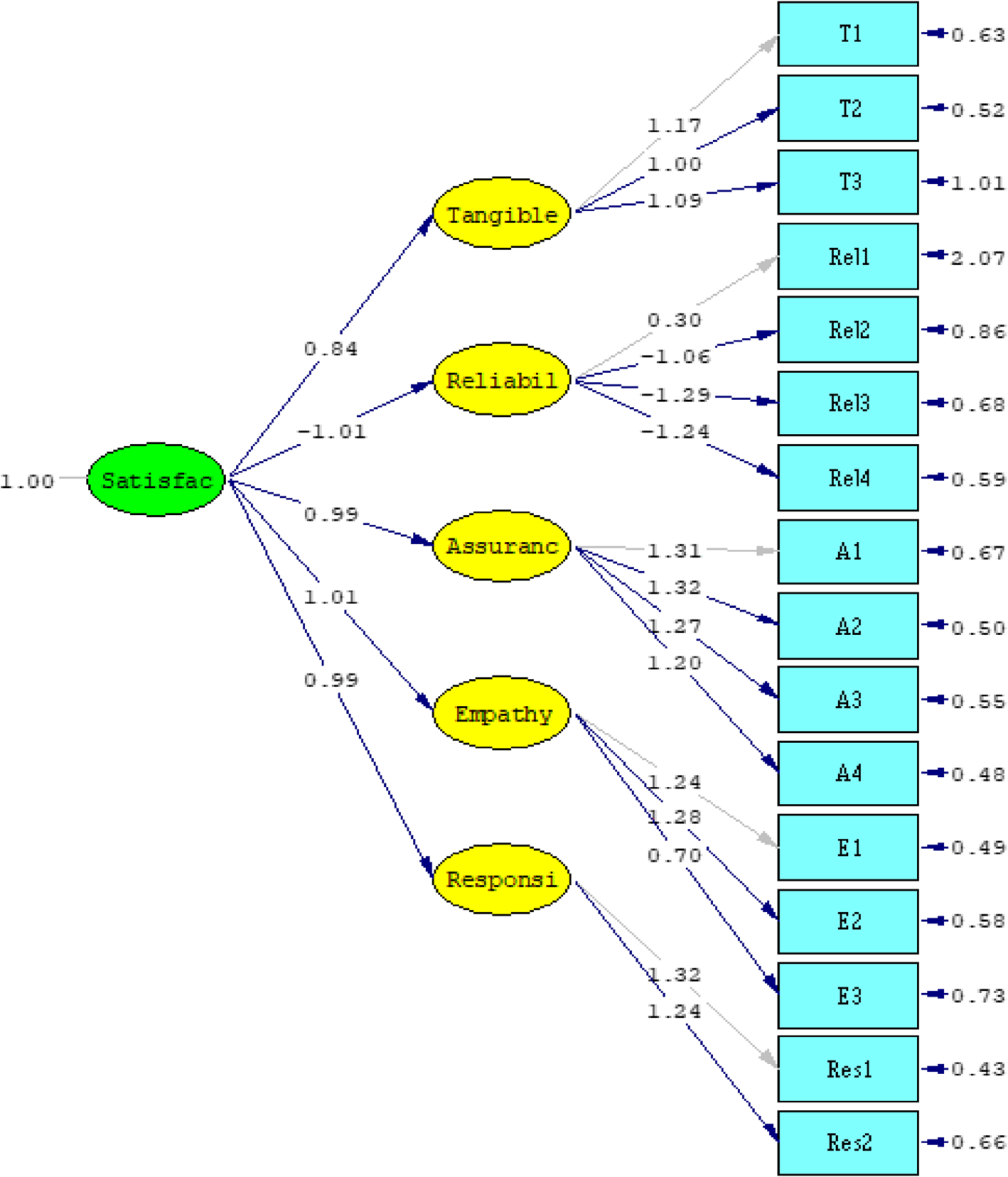
The final output of the factor analysis

To check the validity of the model, it is necessary to check the amount and level of significance of the paths between each of the latent variables with their related indicators. For the confirmatory factor analysis of the entire questionnaire, it is necessary to estimate the significant coefficients of t and estimate the standard factor loadings. Factor loadings show the weight of each question in the level of satisfaction. To find the weight of each question, the method of confirmatory factor analysis and Liesel software was used to check the appropriateness of the general model of the questionnaire in addition to using factor loadings as the weight of the questions. The strength of the relationship between the factor (hidden variable) and the observable variable (questionnaire questions) is shown by the factor load. The final output of the factor analysis is shown in Fig. 2. All variables show a high correlation with their respective constructs.

An important point to note is that the fit of the model should be evaluated through different methods and criteria to check its fit from different dimensions. One of the main indicators is the ratio of chi-square to degrees of freedom, also known as relative chi-square, in which values less than 3 are interpreted as very good. Another main index that is examined is the Root Mean Square Error of Approximation (RMSEA), which emphasizes the error of approximation that values less than 0.1 indicate an acceptable fit of the model. Some sources recommend the use of Non-Normed Fit Index (NNFI) and Comparative Fit Index (CFI) to check model fit. Also, other indices such as Normed Fit Index (NFI) and Incremental Fit Index (IFI5) can also be used to check the fit of the model.

Some of the important indicators resulting from the factor analysis and the target range for their acceptance [3], are shown in Table 2. As can be seen, the analyzed indicators show the appropriateness of the model.

**Table 2:** Confirmatory factor analysis model estimation results

Indicator	$\frac{{\chi }^{2}}{df}$	RMSEA	NFI	NNFI	CFI	IFI
Acceptable domain	Smaller than 3	Smaller than 0.1	Greater than 0.9	Greater than 0.9	Greater than 0.9	Greater than 0.9
Model estimation	1.70	0.094	0.98	0.98	0.98	0.98

The results of the table and the figure show the approval of all the routes in the conceptual model of SERVQUAL for customers. In other words, all the factors mentioned in the conceptual model have an effect on the response variable, which is the level of customer satisfaction.

In order to perform further statistical analysis, the normality of the data frequency distribution is first checked by means of the Kolmogorov-Smirnov test. If the data has a normal distribution, it is possible to use parametric tests. In this paper, due to the non-normality of the variables, non-parametric tests have been used. It is worth mentioning that in all tests, p-value less than 0.05 indicates the significance of the corresponding test and the rejection of the null hypothesis.

Since the distribution of the data in the state of perception and expectation was non-normal, the difference between the average of the current and desired conditions of customers is checked using the Wilcoxon test. If the assumption of equality between perceptions and expectations is confirmed, the hypothesis of customer satisfaction with the services provided can be accepted, otherwise, the hypothesis of customer satisfaction with the services received is rejected. It should be noted that the Wilcoxon test was performed both to check the main hypothesis of the research and to check the sub-hypotheses, the results of which are shown in Table 3. According to the considered significance level, which is equal to 0.05, if the significance level is less than 0.05, the test is significant. Considering that the null hypothesis is the equality of the averages and the opposite hypothesis is the inequality of the averages of perceptions and expectations in the SERVQUAL model, the significance of the test means that the null hypothesis is correctly rejected.

**Table 3:** The results of the Wilcoxon test on research hypotheses

Hypothesis	Test statistic	*p*-value	Decision
Customers are satisfied with the tangible dimensions of insurance companies in Iran	-16.115	0.000	Reject the null hypothesis
Customers are satisfied with the reliability of insurance companies in Iran	-20.092	0.000	Reject the null hypothesis
Customers are satisfied with the assurance of insurance companies in Iran	-19.794	0.000	Reject the null hypothesis
Customers are satisfied with the empathy of employees of insurance companies in Iran	-18.739	0.000	Reject the null hypothesis
Customers are satisfied with the responsiveness of the employees of insurance companies in Iran	-19.477	0.000	Reject the null hypothesis
The main hypothesis is: Customers are satisfied with the quality of supplementary health insurance services provided by insurance companies in Iran	-21.156	0.000	Reject the null hypothesis

According to the results of the Wilcoxon test reported in Table 2, there is a significant difference between the perceptions and expectations of customers in all dimensions. In other words, the rating of customers' perceptions of service quality (existing situation) was significantly lower than their expectations, and in other words, there is a negative gap in all five dimensions. The results show that customers are not satisfied with the overall quality of the services received.

## Conclusion

In this research, the level of satisfaction of policyholders with supplementary health insurance in Iran was investigated. The findings of the current research showed that there is a negative and significant gap in all dimensions of service quality. The negative gap shows that from the point of view of the customers, the service provision is not up to their expectations and necessary measures should be taken to meet the expectations of the customers. A similar result was obtained by [8]. To improve the situation, they recommended that the transactions of insurance companies should be transparent and honest. Electronic services should be provided to customers. Employees should be polite. The cost of services should be reduced. Also, the results of [12], showed that policyholders generally are not satisfied with the Life insurance products. To increase satisfaction, they suggested further research on the key factors affecting customer satisfaction. The results of [5] were slightly different. In this research, there was not a big gap between expectations and perceptions in the tangible dimension. This issue is due to the different nature of life insurance. Because most of the services to the insurance policyholders are in absentia. The biggest gap in this research was in the dimension of empathy.

Insurance companies should identify the factors affecting the dissatisfaction of policyholders with complementary health insurance services in order to reduce the churn of customers by eliminating them.

To reduce the gap in the dimension of empathy, a sense of compassion and respect and special attention to each customer should be included in the company's plans, and training and encouraging employees to deal with customers correctly can be one of the most important and effective measures in this field. In terms of reliability, performing the services promised to the policyholders in a certain time, making corrections at the first opportunity, recording the records of the policyholders without errors, and informing the policyholder of the exact time of doing the work helps to reduce the gap.

In terms of responsiveness and assurance, the employees' behavior with the policyholders should be such that over time the policyholders have confidence and trust in the company. The behavior of the employees with the policyholders should always be respectful and the employees should be trained in such a way that they have enough knowledge to answer the questions of the policyholders.

Among other factors that contribute to the improvement of the quality of services in supplementary health insurance, it is possible to mention the revision of the insurance processes when customers benefit from insurance services so that by reducing the administrative bureaucracy, the benefits of supplementary medical insurance can be used better. The use and promotion of offline services also prevent frequent visits of insurance policyholders and create more satisfaction for them.

In future research, it is possible to use several customer satisfaction measurement models in supplementary medical insurance and compare the results. It also carried out customer satisfaction measurement for other insurance fields in Iran.

## Data Availability

All raw data and also the file of this study have been prepared in Persian (not English). But the corresponding author will gladly provide any supporting materials upon request.
